# The Generation of Therapeutic Fc Fusion Proteins to Trap Both IL-4 and IL-13

**DOI:** 10.14789/ejmj.JMJ25-0070-OT

**Published:** 2026-03-19

**Authors:** SHUNICHI MIYAZAKI, AYAKO KAITANI, KUMI IZAWA, TOMOAKI ANDO, AKIE MAEHARA, NOBUHIRO NAKANO, HIDEOKI OGAWA, KO OKUMURA, JIRO KITAURA

**Affiliations:** 1 Atopy (Allergy) Research Center, Juntendo University Graduate School of Medicine, Tokyo, Japan; 1 Atopy (Allergy) Research Center, Juntendo University Graduate School of Medicine, Tokyo, Japan; 2 Juntendo University School of Medicine (6th year medical student), Tokyo, Japan; 2 Juntendo University School of Medicine (6th year medical student), Tokyo, Japan

**Keywords:** type 2 inflammation, IL-4, IL-13, cytokine trap, Fc fusion protein

## Abstract

mIL-13Rα1-mIL-4Rα-hFc protein efficiently suppressed signaling of both IL-4 and IL-13. Accordingly, the drug to efficiently trap both cytokines may be promising as alternative medicine.

## Background

Type 2 inflammation induced by type 2 cytokines such as IL-4 and IL-13 is a major driver of allergic diseases. The type I IL-4 receptor (type I IL-4R) is a heterodimer of IL-4Rα and IL-2Rγc. On the other hand, the type II IL-4R is a heterodimer of IL-4Rα and IL-13Rα1, which also acts as IL-13R. The anti-IL-4Rα antibody dupilumab, which can inhibit signaling of both IL-4 and IL-13, is effective for allergic diseases^1)^.

## Methods

The C-terminus of the extracellular domain of mouse IL-4Rα was fused to the N-terminus of the Fc portion of human IgG1 to generate mIL-4Rα-hFc protein, whereas the C-terminus of the extracellular domain of mouse IL-13Rα1 was fused to the N-terminus of mIL-4Rα-hFc protein to generate mIL-13Rα1-mIL-4Rα-hFc protein. A murine keratinocyte cell line Kera-308, expressing type II IL- 4R, was stimulated with IL-4 or IL-13 in the presence of these Fc fusion proteins, including hFc alone, to measure levels of CCL11 released using ELISA.

## Results

mIL-4Rα-hFc inhibited CCL11 release from Kera- 308 cells stimulated with IL-4, but not with IL-13, whereas mIL-13Rα1-mIL-4Rα-hFc inhibited that stimulated with IL-4 and with IL-13.

## Conclusions

**Figure 1:**
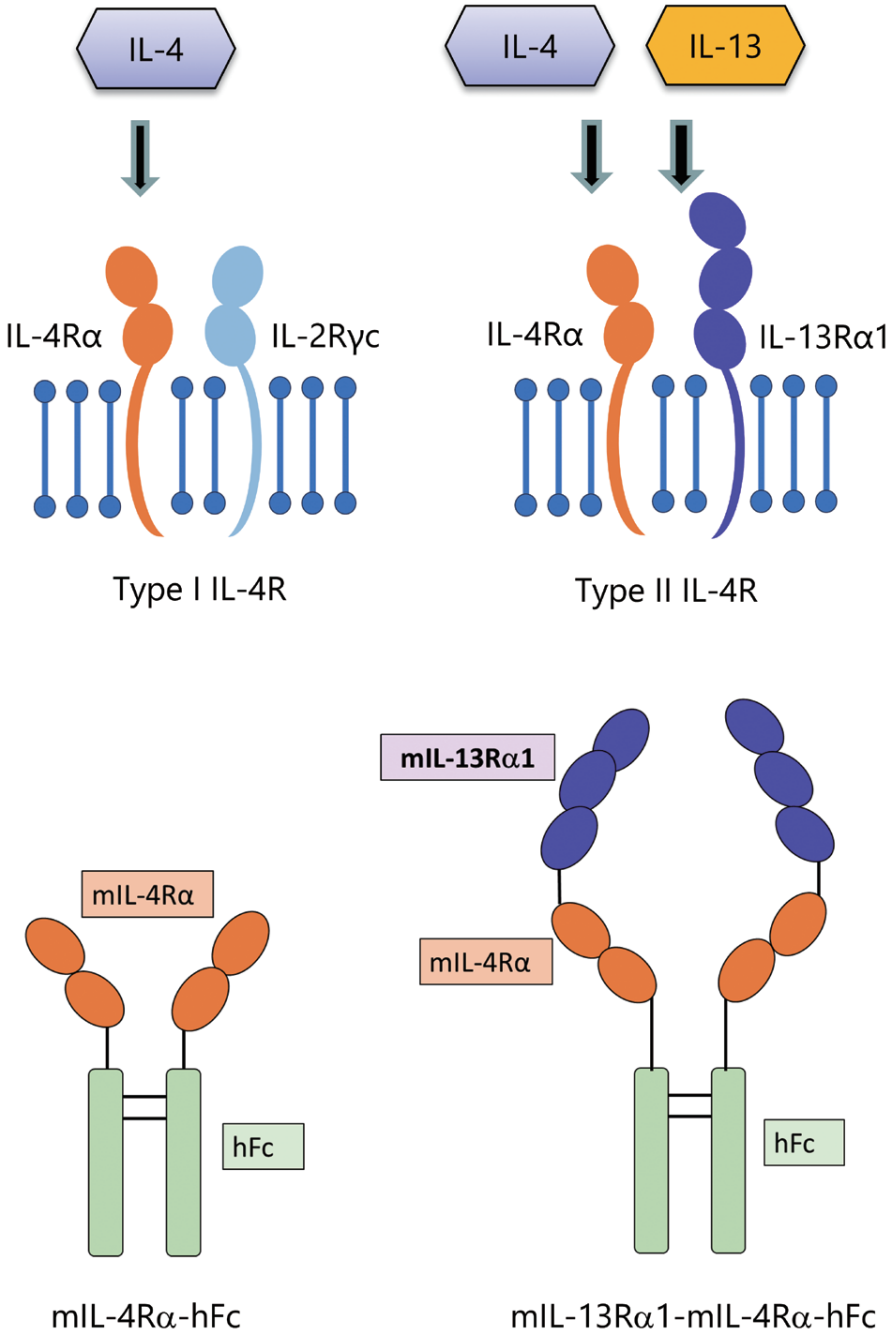
Schematic representation of type I IL-4R, type II IL-4R, and Fc fusion proteins mIL-4Rα-hFc and mIL-13Rα1-mIL-4Rα-hFc

mIL-13Rα1-mIL-4Rα-hFc protein efficiently suppressed signaling of both IL-4 and IL-13 (Figure 1). Accordingly, the drug to efficiently trap both cytokines may be promising as alternative medicine.

## Author contributions

SM performed all the experiments and participated in writing the manuscript. AK assisted with the in vitro experiments, analyzed the data, and actively participated in manuscript writing. KI, TA, AM, and NN assisted with the in vitro experiments. HO and KO analyzed the data. JK conceived the project, analyzed the data, and actively participated in manuscript writing. All authors contributed to the article. All authors read and approved the final manuscript.

## Conflicts of interest statement

The authors declare that there are no conflicts of interest.
